# Supplementary material for: Separating phases of allopolyploid evolution with resynthesized and natural Capsella bursa-pastoris
Source: eLife. 2024 Jan 8;12:RP88398. doi: 10.7554/eLife.88398 (PMC10945474; doi:10.7554/eLife.88398)
Supplement: Figure 1—source data 1. [file elife-88398-fig1-data1.docx]

**Figure 1–Source Data 1** *Capsella* plants used in the present study

| **Species** |  | **Line ID** | **Line notation** | **Latitude (N)** | **Longitude (E)** | **Genetic cluster*** | **Reference** |
| --- | --- | --- | --- | --- | --- | --- | --- |
| *Capsella bursa-pastoris* |  | BEL5 | Cbp-6 | 50.55 | 128.28 | EUR | this study |
|  |  | JO56 | Cbp-11 | 31.97 | 35.98 | ME | this study |
|  |  | SE14 | Cbp-8 | 62.64 | 17.94 | EUR | this study |
|  |  | TBS195 | Cbp-3 | 33.57 | 107.45 | ASI | this study |
|  |  | TR83 | Cbp-12 | 41.02 | 28.97 | ME | this study |
|  |  | TY118 | Cbp-4 | 37.55 | 112.32 | ASI | this study |
| *Capsella grandiflora* |  | 81 | - | 37.30 | 22.06 | - | (Duan *et al.*, 2023) |
| *Capsella orientalis* |  | URAL-RUS5 | - | 55.11 | 61.39 | - | (Duan *et al.*, 2023) |

* The three major genetic clusters (populations) of natural *C. bursa-pastoris* were Asian (ASI), Middle-East (ME), and Europe (EUR), defined by Kryvokhyzha *et al.* (2016, 2019).
