# Supplementary material for: Separating phases of allopolyploid evolution with resynthesized and natural Capsella bursa-pastoris
Source: eLife. 2024 Jan 8;12:RP88398. doi: 10.7554/eLife.88398 (PMC10945474; doi:10.7554/eLife.88398)
Supplement: Figure 2—source data 1. [file elife-88398-fig2-data1.docx]

**Figure 2–Source Data 1** Effects of plant group and positions (tray ID) on phenotypes

|  |  | **General linear models,**  **ANOVA (type III tests)** | | | | | | | |
| --- | --- | --- | --- | --- | --- | --- | --- | --- | --- |
|  |  | **~ Group** | | |  | **~ Group + Tray_ID** | | | |
|  |  | **df** | **F** | **p-value** |  | **df** | **F** | **p-value** | |
| Petal length | Group | 4 | 296.0 | **<2.2e-16** |  | 4 | 310.81 | **<2.2e-16** | |
|  | Tray | - | - | **-** |  | 35 | 1.78 | **0.0111** | |
|  | Residuals | 160 | - | **-** |  | 125 | - | **-** | |
| Petal width | Group | 4 | 384.25 | **<2.2e-16** |  | 4 | 388.11 | **<2.2e-16** | |
|  | Tray | - | - | - |  | 35 | 1.12 | 0.321 | |
|  | Residuals | 160 | - | **-** |  | 125 | - | **-** | |
| Sepal length | Group | 4 | 194.08 | **<2.2e-16** |  | 4 | 218.73 | **<2.2e-16** | |
|  | Tray | - | - | **-** |  | 35 | 1.65 | **0.0242** | |
|  | Residuals | 160 | - | **-** |  | 125 | - | **-** | |
| Sepal width | Group | 4 | 187.15 | **<2.2e-16** |  | 4 | 192.74 | **<2.2e-16** | |
|  | Tray | - | - | - |  | 35 | 1.18 | 0.247 | |
|  | Residuals | 160 | - | **-** |  | 125 | - | **-** | |
| Pistil length | Group | 4 | 95.35 | **<2.2e-16** |  | 4 | 96.24 | **<2.2e-16** | |
|  | Tray | - | - | - |  | 35 | 1.18 | 0.253 | |
|  | Residuals | 160 | - | **-** |  | 125 | - | **-** | |
| Pistil width | Group | 4 | 78.47 | **<2.2e-16** |  | 4 | 74.67 | **<2.2e-16** | |
|  | Tray | - | - | - |  | 35 | 0.879 | 0.663 | |
|  | Residuals | 160 | - | **-** |  | 125 | - | **-** | |
| Stamen length | Group | 4 | 173.99 | **<2.2e-16** |  | 4 | 190.61 | **<2.2e-16** | |
|  | Tray | - | - | - |  | 35 | 1.46 | 0.069 | |
|  | Residuals | 160 | - | **-** |  | 125 | - | **-** | |
| Stem length | Group | 4 | 84.52 | **<2.2e-16** |  | 4 | 90.71 | **<2.2e-16** | |
|  | Tray | - | - | - |  | 35 | 1.33 | 0.128 | |
|  | Residuals | 166 | - | **-** |  | 131 | - | **-** | |
| Flowering time | Group | 4 | 49.20 | **<2.2e-16** |  | 4 | 25.64 | **1.13e-15** | |
|  | Tray | - | - | - |  | 35 | 1.37 | 0.104 | |
|  | Residuals | 165 | - | **-** |  |  | - | **-** | |
| Pollen grains per flower | Group | 4 | 164.65 | **<2.2e-16** |  | 4 | 145.12 | **<2.2e-16** | |
|  | Tray | - | - | - |  | 35 | 0.91 | 0.614 | |
|  | Residuals | 137 | - | **-** |  | 102 | - | **-** | |
| Number of seeds in 10 fruits | Group | 4 | 152.54 | **<2.2e-16** |  | 4 | 170.78 | **<2.2e-16** | |
|  | Tray | - | - | - |  | 35 | 1.59 | **0.0367** | |
|  | Residuals | 146 | - | **-** |  | 111 | - | **-** | |
|  |  | **Generalized linear models (quasibinomial, link=“logit”),**  **ANOVA (type III tests)** | | | | | | | |
|  |  | **~ Group** | | |  | **~ Group + Tray_ID** | | | |
|  |  | **df** | **F** | **p-value** |  | **df** | **F** | | **p-value** |
| Pollen viability | Group | 4 | 24.39 | **2.97e-15** |  | 4 | 21.06 | | **1.12e-12** |
|  | Tray | - | - | **-** |  | 35 | 0.723 | | 0.862 |
|  | Residuals | 137 | - | **-** |  | 102 | - | | **-** |
| Proportion of normal seeds | Group | 4 | 59.16 | **<2.2e-16** |  | 4 | 83.72 | | **<2.2e-16** |
|  | Tray | - | - | **-** |  | 35 | 1.35 | | 0.121 |
|  | Residuals | 146 | - | **-** |  | 111 | - | | **-** |

-: Not applicable. p-values < 0.05 were highlighted in bold.
