# Supplementary material for: Separating phases of allopolyploid evolution with resynthesized and natural Capsella bursa-pastoris
Source: eLife. 2024 Jan 8;12:RP88398. doi: 10.7554/eLife.88398 (PMC10945474; doi:10.7554/eLife.88398)
Supplement: Figure 4—source data 1. [file elife-88398-fig4-data1.docx]

**Figure 4–Source Data 1** Additive and non-additive gene expression in allotetraploid groups


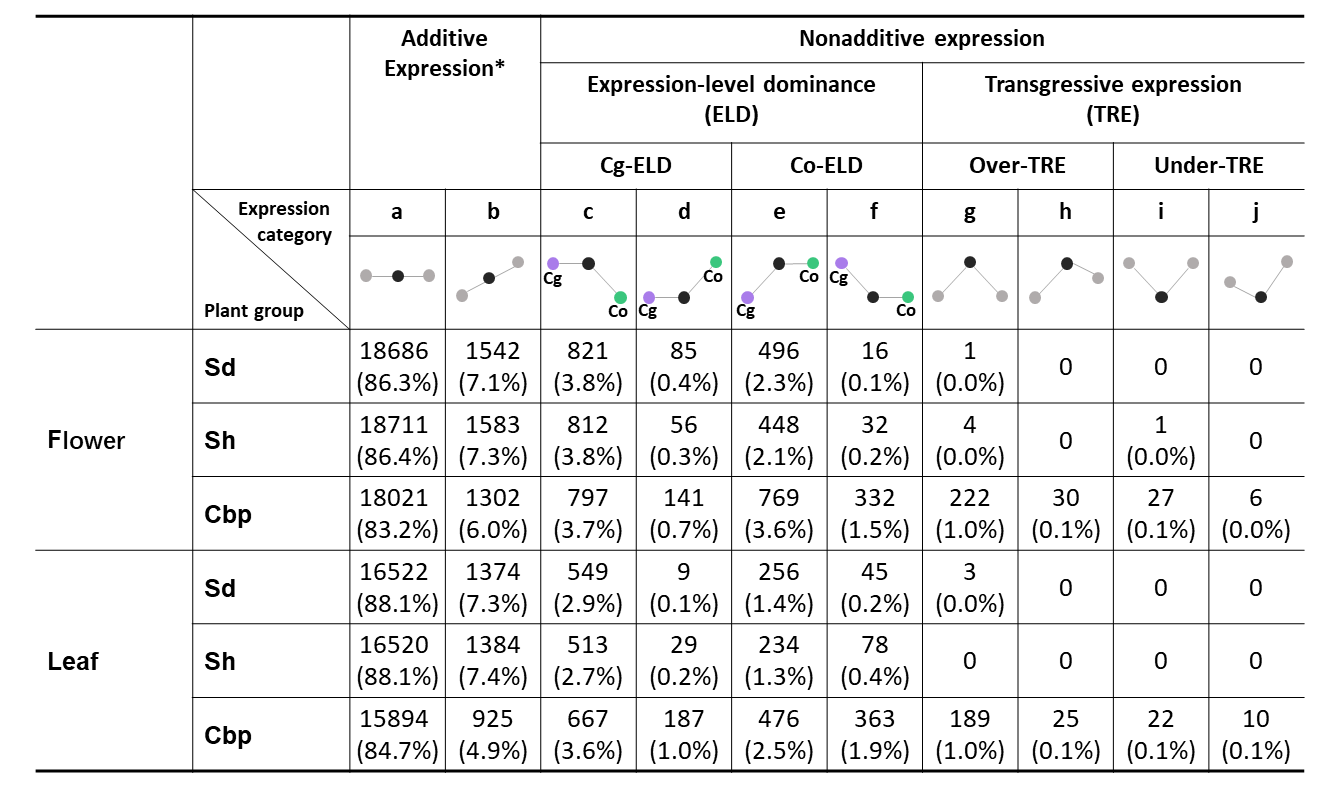


*Partial expression-level dominance (expression level in allotetraploids was not the same as the mid-parent value but still in the middle of the two diploid groups) was included in category b of additive expression. The ten gene expression categories were: a) additive expression with no parental differentiation, b) partial ELD or additive expression with parental differentiation, c) Up-regulated ELD toward diploid *C. grandiflora* (Cg2), d) Down-regulated ELD toward Cg2, e) Up-regulated ELD toward diploid *C. orientalis* (Cg2), f) Down-regulated ELD toward Co2, g) Up-regulated TRE with no parental differentiation, h) Up-regulated TRE with parental differentiation, i) Down-regulated TRE with no parental differentiation, g) Down-regulated TRE with parental differentiation.
