# Supplementary material for: Separating phases of allopolyploid evolution with resynthesized and natural Capsella bursa-pastoris
Source: eLife. 2024 Jan 8;12:RP88398. doi: 10.7554/eLife.88398 (PMC10945474; doi:10.7554/eLife.88398)
Supplement: Figure 8—source data 1. [file elife-88398-fig8-data1.docx]

**Figure 8–Source Data 1** Expression level fold-change (log2FC) of homoeologs relative to the corresponding gene in diploid groups among genes with expression level dominance (ELD) in flowers or leaves.

| **Tissue** | **Source of ELD** | **log2FC of**  **EL-dominant homoeologs (mean**$\boldsymbol{\pm}$**se)** | **log2FC of**  **EL-recessive homoeologs (mean**$\boldsymbol{\pm}$**se)** | **p-value*** |
| --- | --- | --- | --- | --- |
| Flower | Sd/Sh ELD | 0.639$\pm$0.013 | 1.823$\pm$0.034 | <2.2e-16 |
|  | Cbp-specific ELD | 1.208$\pm$0.037 | 2.621$\pm$0.058 | <2.2e-16 |
| Leaf | Sd/Sh ELD | 0.792$\pm$0.024 | 1.933$\pm$0.048 | <2.2e-16 |
|  | Cbp-specific ELD | 1.439$\pm$0.045 | 3.067$\pm$0.072 | <2.2e-16 |

*The difference of expression fold change between EL-dominant and EL-recessive homoeologs was tested by Welch’s two sample t-tests.
